# Impregnation of pectin-cedarwood essential oil nanocapsules onto mini cotton bag improves larvicidal performances

**DOI:** 10.1038/s41598-020-70889-z

**Published:** 2020-08-24

**Authors:** Smriti Kala, Nisha Sogan, S. N. Naik, Amrish Agarwal, Jitendra Kumar

**Affiliations:** 1 Formulation Division, Institute of Pesticide Formulation Technology (IPFT) (Ministry of Chemicals and Fertilizers, Government of India), Gurugram, Haryana 122016 India; 2 Center for Rural Development Technology (CRDT), Indian Institute of Technology (IIT) Delhi, Delhi, 110016 India; 3 National Institute of Malaria Research (NIMR), Delhi, 110077 India

**Keywords:** Nanobiotechnology, Techniques and instrumentation

## Abstract

The use pesticide is one of the indispensable means to combat mosquito borne diseases. However, the repeated use of synthetic pesticides has induced resistance in the vector pest along with undesirable impact on the environment. The biodegradability, non-persistent and user’s safety are the root cause to prefer plant-derived pesticides to synthetic ones. The botanical based pesticides tend to degrade rapidly under the influence of several environmental factors. For the feasible application as pesticides, the plant products are formulated either as liquid or as purely solid. Despite well-established formulation technology in pesticide delivery, their handling trouble is being ignored. There is difficulty in liquid formulation of pesticide products, as they are prone to splashing and spillage, resulting in contamination, wastage and direct exposure to skin; whereas a solid formulation tends to produce dust. In the present work, cedarwood (*Cedrus deodara*) essential oil embedded pectin nanocapsules were produced. The nanocapsules were characterized according to their morphology, size, encapsulation efficiency and thermal stability. Furthermore, the nanocapsules were impregnated onto mini cotton tea bags to be employed as RTU (ready to use) formulation for treating the breeding sites of mosquitoes. The larvicidal activity of the bags treated with pectin-cedar wood nanocapsules was assessed against malaria vector, *Anopheles culicifacies* and 98% mortality was recorded till 4 weeks, this suggests its potential and hassle free applications in controlling mosquito vector.

## Introduction

Global health threats, primarily due to mosquito borne diseases can devastate communities in social and economic terms and hinder development progress^1^. Among the various vector borne diseases, malaria is the major public health issue, which transmits through the bite of *Anopheles* mosquitoes^2^. According to the World Health Organization (WHO) 2019 report, malaria causes 228 million morbidity cases and 405,000 number of mortality^3^. *Anopheles culicifacies* is alone accountable for 60–65% of the malaria burden^4^. A Mosquito-borne disease remains a challenge since there is no vaccine, and further drug resistance is an increasing threat. Under these circumstances, vector control plays a vital role and often remains the only way to prevent disease outbreaks.

The control of malaria vector, *Anopheles,* relies upon indoor residual spray (IRS) and insecticide treated nets (ITN)^5^. Several other control tools have also been proposed including; nano pesticides, microbial pesticides (that are natural occurring bacteria, fungi and viruses) and pesticides of plant origin, which may act as repellents, oviposition deterrents and larvicides etc.^6,7^. A new generation biotechnological tool in mosquito control program, based on genetically modified mosquitoes and sterile Insect Technique (SIT) is also being considered^7,8^. Despite significant progress so far, further investigations are needed to determine if genetically modification based strategies can be an effective method of mosquito control. These facts point out that the use of pesticides may be probable approach to be considered for the reasonable mosquito targeting.

The tackling of the mosquito at the immature stage has been considered more convenient, since the insect is most vulnerable. Moreover, the pesticide application is on selected and defined area, therefore, the contamination and wastage due to pesticide applications could be minimized. In the current scenario the synthetic pesticides are extensively used to control larval stages, which is resulting in resistance development, persistence and toxicity to non-target. In this context, plant derived pesticides may preferred on account of their non-persistent and biodegradability^9^.

The recent advancements have extensively focused on plants based bio-pesticides as an efficient larvicide against *Anopheles culicifacies*. In particular few of botanical pesticides, such as Neem (*Azadirachta indica*) oil, bio-waste of Cashew nut (*Anacardium occidentale*), leaf and seed extracts of castor (*Ricinus communis)*, Paracress *(Spilanthes acmella), Yellow berry night shade (Solanum xanthocarpum), Clove (Syzygium aromaticum*)*,* Shisham *(Dalbergia sissoo*)^10–14^ have shown significant larvicidal activity . Although the use of plant-derived botanical pesticides (essential oil and crude plant extract) has occupied key space in pest management yet the application of botanicals is limited due to poor water solubility and further feasibility of the plant-based product is not assured because of volatility, degradation, and stability. Consequently, the potential use of plant material in large-scale applications is not considered as realistic. To overcome these drawbacks bioactive plant products may be formulated; using polymers, plasticizers, stabilizers and biodegradable additives^15^. The additives in formulating a plant product may include; polymers, emulsifying agents, surfactants, solvents, stabilizers, de-foamers which ensure the stability, adherence and controlled release of the bioactive compounds, depending on the type of formulation^16^.

Himalayan Cedar (*Cedrus deodara*) is large evergreen coniferous tree, found abundantly grown throughout the western Himalayas regions of India. The essential oil of cedar wood (CWO) has been reported to possess insecticidal activities against stored pests, houseflies and lepidopteran insects^17^. The extraction method is one of prime factors that determine the quality of essential oil since; inappropriate extraction procedure can lead to the damage or alter the constituents of essential oil. Extraction of essential oils can be carried out by various means, as solvent extraction, supercritical extraction and steam distillation. In the present study steam distilled oil was used. Although steam distillation (SD) is the most popular one due to low-cost and easy operation, it would be beneficial to conduct investigation on, the effect of extraction technique on the phyto-constituents of essential oil. The promising results obtained in the preliminary examination with the steam distilled essential oil of cedar wood; against the larva of *Anopheles culicifacies* has encouraged us to use it further.

In recent years, there has been an increased interest in nanotechnology as a means of improving the effectiveness of pesticide, while at the same time minimizing their environmental impacts. Several studies have described the use of nanostructure systems that offer better efficacy as a mosquito larvicide. Some of the most promising nanostructured systems that can be used as larvicides are based on polymeric encapsulation, followed by nanoemulsions and nanogels, etc. Nanoemulsions that can be formulated within the droplet size range of 20–200 nm^18^, and, nanogel that are hydrogel particles in the nanometer range, loaded with *Lippia sidoides* oil has been described as an efficient mosquito larvicide^11,19–21^. Among all the nanostructure systems, nanoencapsulation is one of the well-documented strategies, which can contribute towards sustainable vector management program. It has attracted attention due to the potential for assisting in reduced doses of pesticides while at the same time improving stability and offering controlled release along with decreased water contamination, and less risk to the consumers^22^. Pectin is an inexpensive, non-toxic polysaccharide extracted from citrus peels or apple pomaces, and has been used as a food additive, a thickening agent and gelling agent^23^. Pectin is also used as an encapsulating agent leading to release of encapsulated material at the desired site^24^.

While earlier studies have shown the potential of nano bio-pesticides in different connections, however, the studies describing the convenient way of application by which, pesticides are delivered to their biological targets have not exemplified. Since, the most of the larvicidal products described are liquid based; nano/micro- emulsions and encapsulated suspensions, etc. (Supplementary Fig S1). The liquid formulation of pesticide products may affect the extent of exposure, as they are prone to splashing and occasional spillage, resulting in direct skin contact or indirect contact through clothing contamination^25^. Moreover, the solid formulation tends to produce dust. In the recent years, there has been considerable interest in developing biodegradable, easy to use and effective pesticide delivery systems.

The above evidence provides the rationale behind this study that aimed to devise a technique to get the better of liquid preparation, through the concept of the nanocapsules impregnated mini bag. The impregnation of cotton fabric with the nanocapsules and microcapsules has been reported for long-lasting mosquito repellent and antibacterial effects^26,27^. However, such an approach may certainly be used to larvicidal control program on account of its sustainability. Cotton is made up of cellulose and, therefore, it is easily biodegradable. The cotton based tea bags, therefore, can be used to impregnate pectin-cedar wood nanocapsules.

The study is intended to hypothesize the comparative evaluation on the long-lasting larvicidal efficacy of CWO nanocapsules impregnated bags with that of non-encapsulated bags. The study, therefore, explores a new approach for a safe way of pesticide application, employing botanical and nanoparticles. Here, we present a report describing, design and synthesis of nanocapsules impregnated bag to target immature stages of mosquitoes (illustration-Supplementary Fig S2). Further, we also described long lasting efficacy of nano-encapsulted bag against mosquito larvae. The cured tea bags, impregnated with pectin-cedarwood nanocapsules, could be a RTU (ready to use) formulation to treat mosquito-breeding sites. Such an approach can be effortless and may provide long lasting effects. The nanocapsules impregnated bags may be preferred on account of their safety with respect to reduced direct exposure to the pesticides along with convenience in handling and prolonged effectiveness. This reduces application of pesticides to the target, which may cut costs of the active ingredient used for spray applications.

## Results and discussion

### Composition of CWO oil—GC–MS

A total of 49 compounds from the cedarwood essential oil were identified, which accounted for 100% of its total composition. The GC–MS identified several main chemical compounds in the CWO. The peak area correlated to concentration and was greater for β-himachlene (19.8%-RT-retention time 30.7) followed by α-himachlene (15.38%-RT 28.5), Atlantone (14.0%-RT 41.0), Longifolene (7.4%-RT 29.6), Deodarone (4.63%-RT 38). The other compounds detected as an average percentage included; several other sesquiterpenes and monoterpenes. The gas chromatogram of CWO oil is presented in Supplementary Fig S3 and composition percentage of major and average constituents along with RT is given in Table 1. The *Cedrus deodara* is generally rich in sesquiterpenes-himachalene, which was observed as the most abundant constituent of cedar wood essential oil. The results of our findings are in agreement with the previous reports^17,28^ where, similar trends in oil composition of *Cedrus* were reported. However, variation in abundance of constituents was observed in the essential oil, which may differ according to the part of the plant used for the oil extraction and varies according to the geographical zones, the harvest period and the age of the plant^36^. Plants synthesize several secondary metabolites as bio-actives including terpenes and terpenoids. The essential oils of *Cedrus deodara* from the present investigation is therefore identified as a complex mixture of hydrocarbon terpenes and terpenoids, the majority of the first group consists of monoterpenes and sesquiterpenes, and the second group may consists of oxygenated derivatives of hydrocarbon terpenes i.e. terpenoids^29^. Terpenoids are responsible for notable bioactivity properties^30^ and furthermore, the insecticidal activity of essential oils is reported probably due to terpenes and terpenoids content present in the oil^31^. The major identified constituents sesquiterpenes i.e. β-himachalene and α-himachalene, present in oil were reported to possess insecticidal activity^17^. From the above discussion, it may be deduced that the larvicidal activities of CWO essential oil can be because of the high concentrations of himachalene present in the oil, however, the observed larvicidal activity may also be attributed to the synergy between various components of essential oil^32^. Therefore, the phyoto-constituents analysis briefs about the probable compounds responsible for larvicidal efficacy.

**Table 1:** Constituents of cedar wood essential oil identified by GC–MS.

S/N	Compound identified	RT	Mol.Wt	Composition (%)	S/N	Compound identified	RT	Mol.Wt	Composition (%)
	**Major components**								
1	Αlpha Himachalene	28.5	204	15.38	26	Bergamotene	27.8	204	0.09
2	Longifolene - (V4)	29.6	204	7.4	27	Cadina-1,4-Diene	28.02	204	0.36
3	Beta Himachalene	30.7	204	19.88	28	Geranyl-.Alpha.-Terpinene	28.7	272	0.66
4	Deodarone	38	236	4.63	29	8 Cedren-13-Ol Acetate	29.08	262	0.97
5	Atlantone Trans-, Alpha	41	218	14.0	30	Beta Chamigrene	29.2	204	0.45
	**Average components**								
6	Himachelene 1,4 Diene	29.7	204	1.74	31	AmorphaneCis 4,10 epoxy	29.9	222	0.11
7	Neoisolongifolene, 8,9-Dehydro-	31.5	202	1.36	32	Alpha Ylangene	30.2	204	0.04
8	Himachalene Oxide	34.7	220	1.43	33	Alpha DehydroHimachalene	30.9	200	0.76
9	Himachalol	36.3	222	1.59	34	Isolongifolen, 4,5-Dehydro-	31.1	202	0.06
10	Dihydroatlantone	37	220	2.41	35	Ar-Himachalene	31.7	202	0.42
11	(Z)-Gamma-Atlantone	37.7	218	5.08	36	Bisabolene (Z)-, Alpha-	32.1	204	1.79
12	(E)-Gamma-Atlantone	38.2	218	5.39	37	Spathulenol	32.7	220	0.10
13	Atlantone < (Z)-Alpha- >	38.7	218	3.44	38	Cis-Nerolidol	33	222	0.06
14	Thujopsan-2-Alpha-Ol	36.8	222	1.24	39	OxidoHimachalene	33.2	218	0.41
15	Alpha-Pinene	6.5	136	0.06	40	1H-Inden-1-One, 3a,4,5,6,7,7a-Hexahydro-4,4,7a-Trimethyl-	33.5	178	0.31
16	Limona Ketone	14.6	138	1.34	41	Juniperol	34.3	222	0.58
17	Terpeniol	17.7	154	0.03	42	Caryophyllene Oxide	35	220	1.02
18	Alpha Longipinene	24.1	204	0.14	43	Alpha-Bisabolol	35.4	222	0.13
19	2-Ethyl-3,4-Dimethyl-5-Phenyl-1,2-Oxaborolane	25.7	202	0.56	44	Bergamotene Alpha-, Cis-	35.5	204	0.10
20	Sibirene	26.4	204	0.16	45	Thujopsanone 3-Iso	35.7	220	0.24
21	Longifolene	26.5	204	0.90	46	Alpha.-Ylangene	36.1	204	0.21
22	Vestitenone	26.7	178	0.37	47	Bisabolene	36.5	204	0.27
23	Himachala 2-Diene	27.3	204	0.86	48	Acorenol Beta	39.1	222	0.29
24	4,5-Dehydro-Isolongifolene	27.4	202	0.12	49	Dihydroatlantone (E)	41.5	220	0.77
25	Alpha.-Ylangene	27.6	204	0.20					

*S/N* serial number; *RT* retention time; *Mol.wt* molecular weight.

### Preparation and encapsulation efficiency of pectin-CWO nanocapsules

Essential oils are highly effective as pesticide, but inherent volatile and degradable nature limits their applicability. Nanoencapsulation, which involves coating of active within a polymer, can be a favorable approach to prevent the volatility of essential oil and increase their efficacy^33^. Several studies attempted to formulate effective pesticide using the nanoencapsulation technique; the nanoencapsulation of essential oils of thyme and dill with copper nanoparticles improved antifungal activity^34^, essential oil from *Zanthoxylum rhoifolium* leaves encapsulated in PCL (polycaprolactone) against *B. tabaci* showed that nanoencapsulation is an effective protector of the active-ingredient^35^. Moreover, the enhanced efficacy of garlic essential oil as a pesticide, loaded in PEG nanocapsules was described as compared to free garlic oil^36^. However, the utilization of natural polysaccharides such as chitosan, sodium alginate, starch, pectin, etc. has become more popular due to their non-toxic, biodegradable and biocompatible properties^37^. Pectin is safe and an environment friendly polymer^24^ as a pesticide carrier, therefore, the use of the pectin to load cedarwood essential oil, is acceptable. The CWO nanocapsules were produced via ionic gelation method. Ionic gelation involves the interactions of an ionic polymer with oppositely charged ion to produce hydrogel beads also called as gelispheres, which are spherical cross-linked hydrophilic polymeric entity capable of swelling in water and the release of active is controlled by the polymer relaxation^23^. Pectin forms electrostatically stabilised gel networks i.e. gelispheres with divalent metal cations, usually calcium^38^. The addition of cedar wood oil in pectin solution, during the emulsification process followed by addition of CaCl_2_ at constant mixing produced pectin nanocapsules containing cedarwood oil.

The percent encapsulation efficiency (EE %) i.e. the amount of CWO enclosed into the pectin matrix was determined by the UV–Visible spectrophotometry. The average encapsulation efficiency of 60% was obtained under the optimized conditions and concentration (polymer solution with 1% w/v pectin, cross-linker with 0.5 w/v CaCl2). Since, the encapsulation improves the stability of an essential oil^39^, the obtained value of EE represents an attracting value from the industrial point of view. High encapsulation efficiencies of 87.6–90.6% for sunflower oil encapsulated by ionic gelation using pectin have been previously reported^40^. The encapsulation efficiency may vary and affected by many factors such as, the nature of the oil, polymer concentration and oil polymer ratio, stirring speed, etc^41^. Together, the obtained results and evidences from prior literature, confirmed that the use of pectin for nano-encapsulation can be helpful in enhancing efficacy of bioactive by volatility reduction and also controlled targeted delivery of bioactive^42^, which was further proved from the long-lasting residual effects as observed in larvicidal bioassay.

### Size and morphology

**Figure 1:**
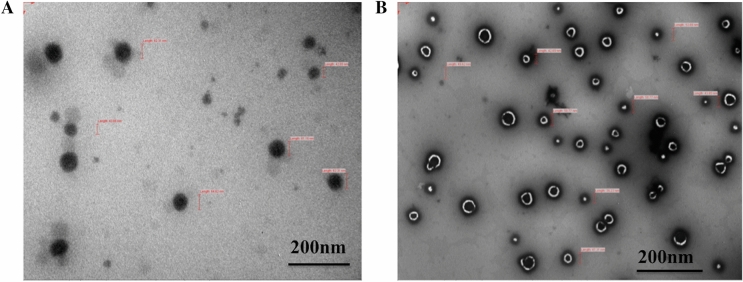
TEM micrographs of (**A**) non-encapsulated cedarwood; (**B**) encapsulated: CWO-loaded pectin nanocapsules.

Size of the nanocapsules in raw state was observed by Transmission electron microscope (TEM). The TEM image of non-encapsulated emulsion shows the droplets with out any wall coating (Fig. 1a). However, TEM images of the pectin nanocapsules loaded with CWO demonstrate regular distribution and spherical shape with size distributed between the ranges of 40 and 80 nm (Fig. 1b). The in-depth morphology of the outer surface of the nanocapsule was perceived which shows the clear presence of the encapsulating-carrier agents having smooth surfaces without any cracks or pores. Since, the essential oils are inherently volatile in nature and the absence of cracks or pores on the surface may signify that the oil-volatiles is protected against degradation and volatility, this is in agreement with the result published by Ali et al.^18^.

### Fourier transform-infrared (FT-IR) spectroscopy

**Figure 2:**
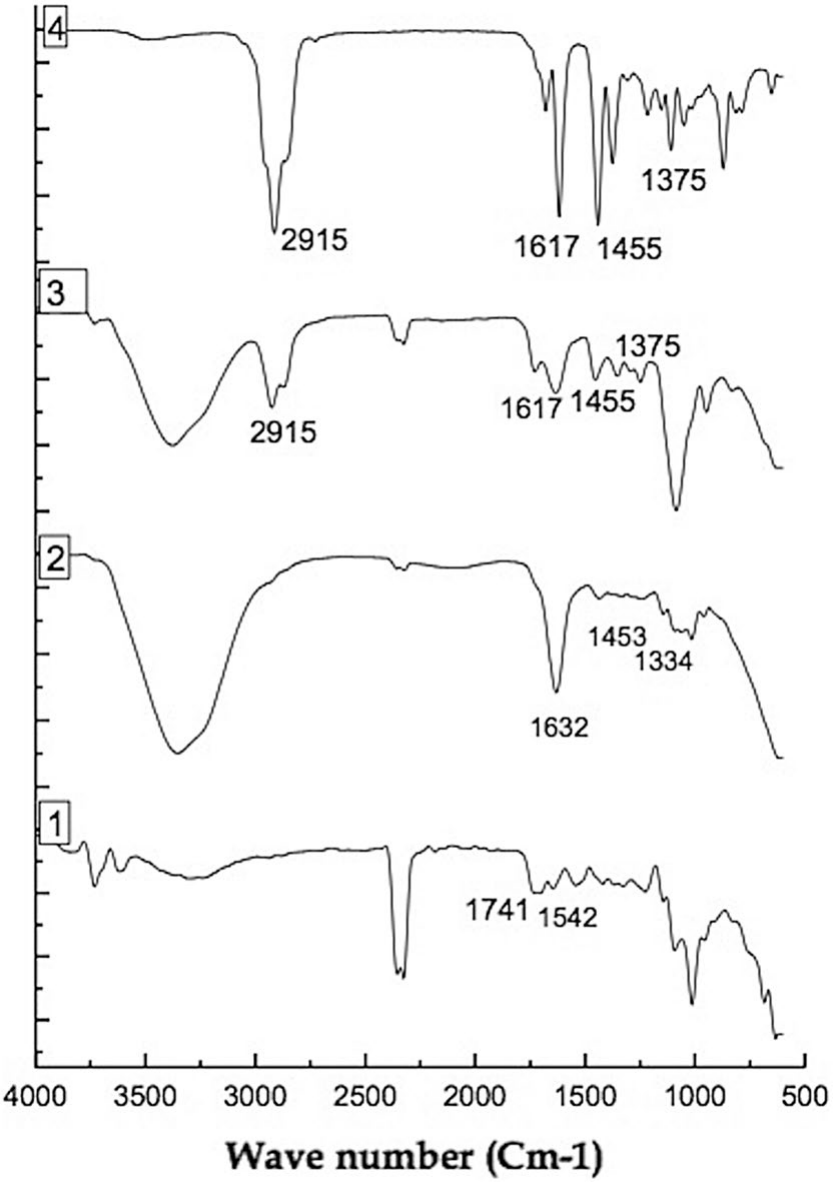
FTIR spectra of (1) pectin powder (2) Blank pectin nanocapsules (3) CWO loaded nanocapsules (4) CWO.

The FTIR spectra of pectin, blank pectin nanoparticles, CWO loaded pectin nanoparticles, and CWO is presented in Fig. 2. The pectin shows one of the characteristic peaks at 1741 cm^−1^, which refers to the CH stretching of carboxyl groups (COOH) and another peak at 1542cm^−1^ attributed to amide group II was observed which is also supported by previous findings^43^. In pectin nanoparticles, the new peak introduces at 1632 cm^−1^, 1,453 cm^−1^ and 1,334 cm^−1^, this might be probably due to intermolecular interaction, which is due to complex formation by electrostatic interaction between pectin and divalent metal cation calcium chloride, the statement is in agreement with previous reports^24,44^. FTIR spectra of CWO showed quite similar general features, with most of the peaks characteristic of oil as reported by Shen et al.^45^. Peaks at 1,375 cm^−1^ and 1455 cm^−1^ are attributed to the methyl, methylene and CH stretch from CWO terpenes. The peaks at 1617 cm^−1^ due to C=C stretching, and 2,915 cm^−1^ due to CH_2_ symmetrical and asymmetrical stretching. After loading of CWO in pectin nanoparticles, in addition to the characteristic peaks of pectin nanoparticles, the peaks of CWO also appear thereby indicating no modification occurs after loading of oil. This change led to an assumption that CWO might be present into the pectin nanoparticles and molecular compatibility between the CWO constituents’ and pectin nanoparticles could exist.

### Thermal properties (DSC)

**Figure 3:**
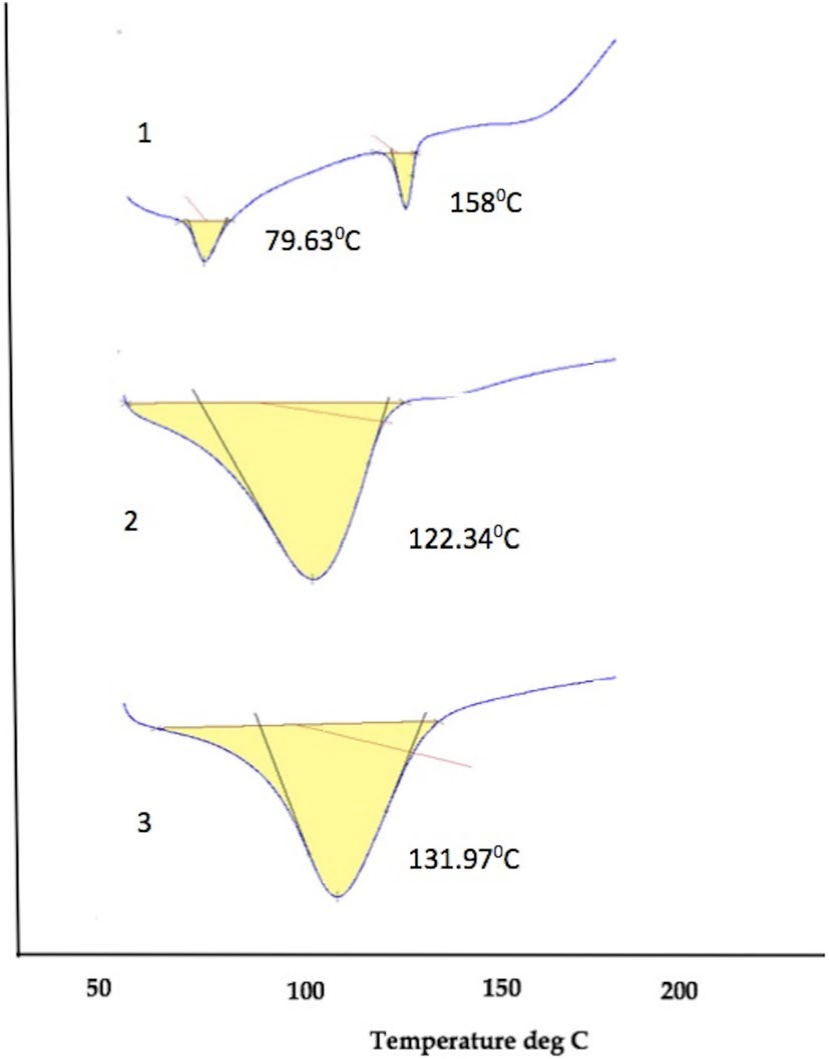
DSC thermograms of (1) pectin powder (2) blank pectin nanocapsules (3) CWO loaded nanocapsules.

DSC aids in providing in depth information about the thermal behavior of various materials, such as melting and crystallization performances^46^. When a molecule interacts with the polymer its melting, boiling or sublimation point generally shifts to a different temperature or disappears within the range^47^. DSC (differential scanning colorimeter) thermograms of pectin, blank pectin nanoparticles, and CWO loaded pectin nanoparticles are presented in Fig. 3. The DSC thermogram of the pectin powder showed a broad endothermic peak at 79 °C which is ascribed to its glass transition temperature and second peak at 158 °C corresponding to its melting point, this is in accordance with previous literature^48^. A broad peak at 122 °C was observed in pectin cross-linked with CaCl_2_. The shifting of the peak at high temperature in cross-linked matrices of pectin revealed high thermal stability compared to their individual components, the statement is in agreement to the previous report^49^. After the loading of cedarwood oil in pectin nanocapsule, the peak further shifts to higher temperature at 131 °C. DSC results obtained here indicated that the CWO was stable in pectin nanocapsules, which was produced by crosslinking with CaCl_2_. The comparable result was reported previously by Chattopadhyay et al.^50^, who used DSC tool to study the compatibility between the polymer, active and other components of the formulations. In both the cases, the shift towards higher temperature was observed in the thermogram of the polymer loaded with active content. Furthermore the thermal stability of nanocapsules at 131 °C after loading with CWO oil points out that the bags can be cured at 100 °C, the statement is consistent with the previous reports published by Liu et al.^51^.

### Coating of capsules on tea bag

The mechanism of adhesion of the pectin nanocapsules on the cotton bag surface in the presence of acrylate may be attributed to surface modification of cotton fibers, illustrated in a supplementary figure (Supplementary Fig S4). During curing the process at 100 °C, there might be an interaction between –OH group of cellulose (Cell-OH) and –COOH group of acrylate, which may lead to formation of ester linkage. The statement is according to previous reports ^26,52^. The ester linkage might be responsible for deposition of pectin -CWO nanocapsules on cotton-bag fibers.

### Surface morphology of coated bags

**Figure 4:**
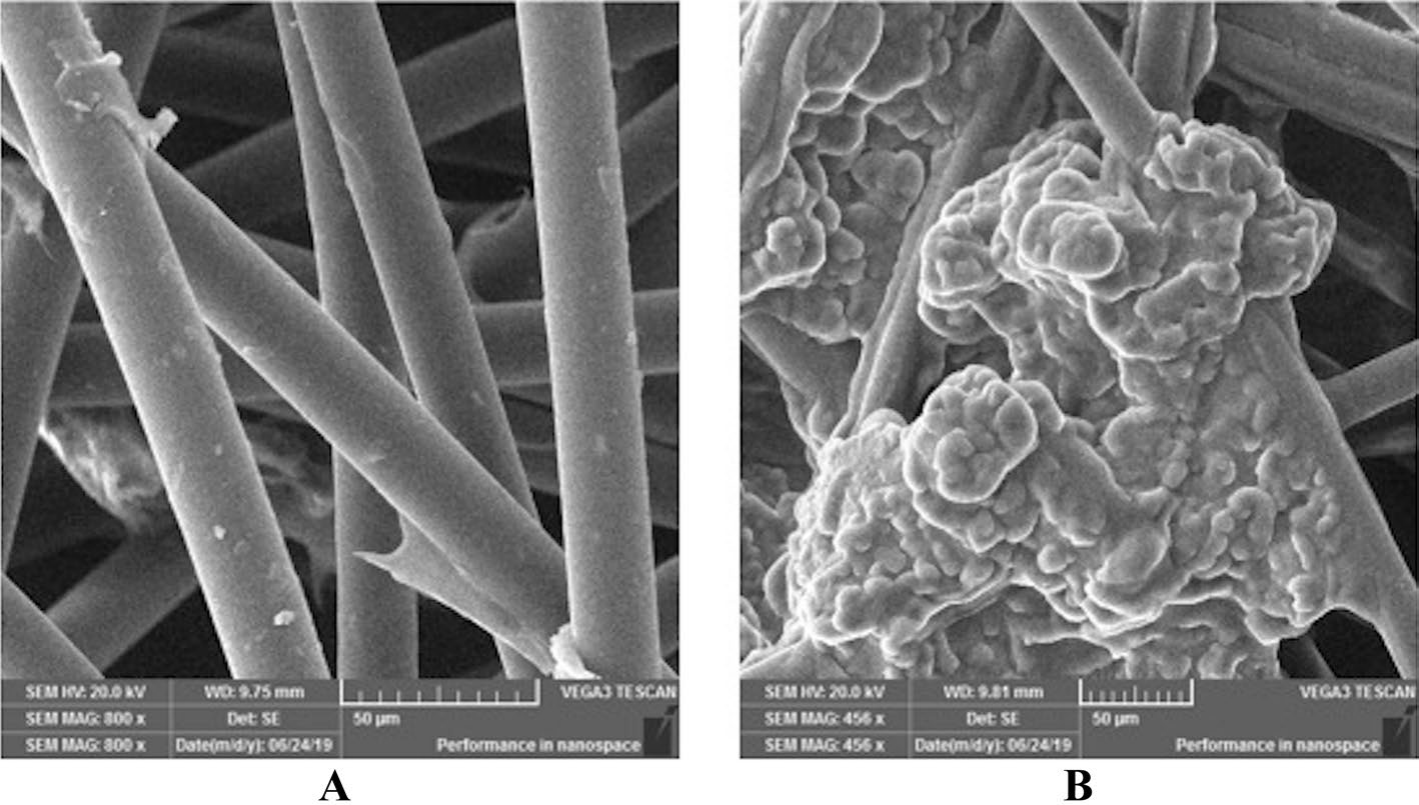
Surface morphology of (**A**) blank bag (**B**) bag impregnated with CWO loaded nanocapsules.

The surface morphology of the bag impregnated with nanocapsules was investigated by SEM. The comparative SEM images of bags before and after impregnation with nanocapsules are presented in Fig. 4. The untreated bag (Fig. 4a) shows blank fibers, whereas, the impregnated bag shows clear presence of nanocapsules onto cotton fibers (Fig. 4b). In addition, the nanocapsules were fixed in the spacing of fibers this may be because of the esterification reaction occurred between acrylate and cellulose during the curing process. The SEM result indicates that the capsules were successfully impregnated on the fibers of bags. A previous study also suggested that the successful impregnation of nanocapsules onto cotton fabric can be carried out using polymeric binder or resin and further its confirmation by SEM imaging^26,52^.

### Residual larvicidal assay

The results of larvicidal bioassay are presented in Table 2. In the larvicidal bioassay; the larvae treated with the encapsulated bag initially exhibited lower larval mortality, which was recorded at 70% (Table 2). The reason for initial lower mortality may be due to slow release of oil contents as owned by pectin nanocapsules. Generally, the release of active from swellable polysaccharides like pectin occurs by either single or by a combination of three different mechanisms: an osmotically driven force, a diffusion mechanism, or erosion or degradation of the polymer. Here in this case the release from pectin nanocapsules probably governed by a diffusion mechanism which includes several phases; penetration of water into the capsule, resulting in swelling of the matrix followed by conversion of the polymer into a rubbery swollen matrix, resulting in release of the active from the swollen matrix, the statement is supported by previous literature^53^. The initial low mortality rate may be attributed to the ascribed slow release mechanism involving diffusion. However, after complete swelling is achieved, the pectin nanocapsules shows burst release, which is justified by increased release of CWO components resulting in increased mortality rate and 90% reduction in larval population up to 2 weeks followed by 98% mortality up to the fourth week. The results may be attributed to the successful encapsulation and the impregnation of CWO nanocapsules on the fiber of the bag, which offered slow release. Contrast to this the bag coated with free emulsion (non encapsulated), there was a constant decline in mortality rate after 2 weeks, which may be due to the free oil that got completely released from the bag initially. However, no mortality was observed in control (distilled water + surfactants). The difference in bioassay of the encapsulated bag and non-encapsulated bag may be because of the thermodegradation and volatility of bioactives of CWO, which may be responsible for losses of oil components in non-encapsulated bag. However, the losses due to the similar factors seem to be prevented by encapsulation process in the encapsulated bag. It has been stated previously that essential oils are volatile and sensitive to environmental factors, nanoencapsulation may prevent their degradation and volatility^54^, hence the long-term efficacy was attained up to 4 weeks with 98% mortality rate may be attributed to nanoencapsulation. There have been evidences indicating the nanoencapsulation process-enhancing efficacies of essential oils^55–57^. Oregano essential oil loaded-chitosan nanoparticles produced thermally stable nanocapsules that showed more effectiveness against fungus *Alternaria alternate* which causes great damages to the post-harvest crops^58^ and zein (corn protein) nanoparticles loaded with neem oil was found more effective and safe to the non-target organisms^59^. Our findings are corroborated by these previous literatures. Further, the results point out that the nanocapsules impregnated bags may be suitable to achieve long-term effect for targeting the breeding sites of mosquitoes.

**Table 2:** Comparative efficacy of encapsulated bag and non-encapsulated bag formulation showing percentage mortality with residual effect on *Anopheles *(± SD standard deviation).

Days	Nano encapsulated		Free oil ( non-encapsulted)	
	Mean No. of dead larva (n = 20)	Reduction in larval population (%)	Mean No. of dead larva (n = 20)	Reduction in larval population (%)
1	14.00 b	70.00 b	19.33 a	96.67 a
2	15.33 b	76.67 b	19.67 a	98.33 a
5	18.00 a	90.00 a	16.67 b	83.33 b
14	19.33 a	96.67 a	9.67 c	48.33 c
21	19.33 a	96.67 a	8.33 c	41.67 c
28	19.67 a	98.33 a	2.33 d	21.67 d

Values (means of 3 replicates). Means (± SD) followed by the same letters (a–d) within columns indicate no significant difference (p < 0.05) from each other in a (Duncan’s multi-range test).

### Histopathological alterations in larva tissues

**Figure 5:**
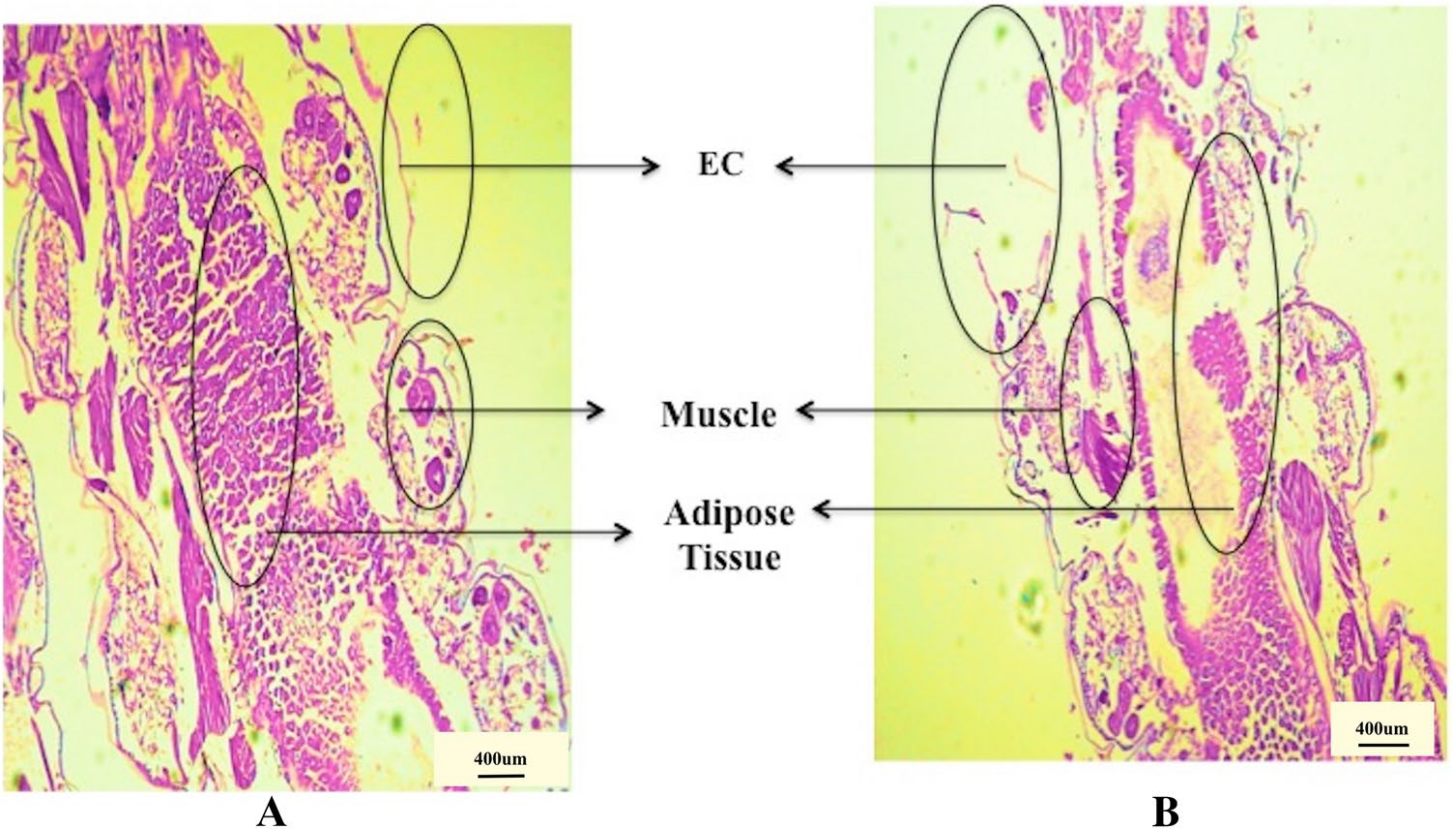
Cytomorphology of midgut of larva (**A**) Control (**B**) CWO nanocapsules, magnification 40×.

Histopathological alterations in the midgut of larva post exposure to bioactive contribute to a better understanding in the possible mode of action of particular bio-pesticide against a larva^60^. Since, the midgut is composed of muscle (M), Adipose tissue (AT) and epidermal cells. (EC). EC is responsible for the production of enzymes and cuticular oxidation process^61^ and have several diverse functions, such as ionic and osmotic regulation, lipid and carbohydrate storage, midgut lumen pH control, digestive enzyme secretion and nutrient absorption^62^. The 4th instar larvae of *An. culicifacies* exposed to nano-CWO showed severe morphological deformities. CWO-nanocapsules directly acted upon, muscle (M), Adipose tissue (AT) and epidermal cells (EC) and displayed completely damaged epithelial cell, muscles and adipose tissue (Fig. 5b). In contrast, LS examination of the control larval tissues displayed intact and undamaged assembly of epithelial cell, muscles and adipose tissue (Fig. 5a). The results are in accordance with previous reports^61^. The toxicity of the plant essential oil (EO) as an insecticide mainly depends on penetration rate of EO into the insect body, their interaction with specific target enzymes and the detoxification ability of the mosquito at each developmental stage^62^. In the present study, the 4th instar larvae *of An. culicifacies* exposed to nano-CWO showed severe morphological deformities. Some earlier researchers have also reported the morphological aberrations induced by nano based botanical pesticide on mosquito larvae^11,63^. The comparatively severe damage occurred in the larval midgut cells treated with nano-CWO may attributed to the remarkable feature of nanoparticles; larger surface and smaller size, which can promote easy penetration into the larval body and disturb their normal cycle of life by interrupting the intake of feed, cell division and breathing, the statement is supported by previous literature^61,64^.

## Chemicals and materials

Pectin (30,000–10,0000 MW) and Calcium chloride were purchased from SRL chemicals, India. Tween80 and Span 20 were purchased from SDFCL India. Cedar wood essential oil and Cotton tea bags were purchased from local supplier. Acrylate (Thickener/Binder aqueous emulsion of acrylic polymer) obtained from Lamberti, Italy.

## Methodology

### Chemical composition of cedarwood oil-(GC–MS)

The major components of cedar wood essential oil were identified through GC–MS (Shimadzu QP 2010 74707 Plus) equipped with an FID and capillary column (0.32 mm i.d., length: 30 m, film thickness 0.25 µm). Injection volume was 1 µl, oven temperature was programmed with 50 °C (hold time 5 min), which was increased at the rate of 10 °C/min to 300 °C (hold time 5 min). Injection temperature was 250 °C, with a split ratio of 10. The chemical constituents of oil were identified using GC–MS by comparing the individual mass spectra with the database of National Institute of Standards and Technology (NIST12 or NIST62-) libraries.

### Preparation and encapsulation efficiency of CWO loaded pectin-nanocapsule

Pectin nanoparticle was prepared via ionic gelation method using calcium chloride as cross-linker, a method described previously with slight modification^24^. Briefly, 1% solution of pectin was prepared, Tween 80 (5): Span80 (2) was added to the solution and was stirred in magnetic stirrer at 1500 ppm. While stirring cedar wood oil was poured to the solution and it was further stirred for 10 min. Finally 0.5% w/v added drop-wise as a cross-linker.

The total content of encapsulated cedarwood essential oil (CWO) was determined spectrophotometrically according to previously described method^65^. Briefly, a fixed weight of microcapsules (200 mg) was taken and diluted with ethanol. The mixture of ethanol and sonicated for 15 min to allow complete breakage of nanocapsules followed by centrifugation for 5 min. The supernatant was collected and the total amount of encapsulated cedarwood essential oil was determined by UV–Vis spectrophotometer at a wavelength of 326 nm using a Labomed UVD 2960 spectrophotometer. For standard curve several dilutions of oil was prepared and curve was constituted. The trend line was obtained by linear regression with standard equation (*Y* = 18.75*X*  − 0.0019) and *R*^2^ value (0.99852). The amount of CEO was calculated by appropriate calibration curve of free CEO in ethanol (R^2^ = 0.999). The encapsulation efficiency of cedarwood essential oil into pectin‐nanocapsules was calculated by using the following equation.

$$EE \left(\%\right)=\frac{\left(WT-WS\right)\times 100 }{\left(WT\right)}$$

(where WT = the total amount of cedarwood used in the encapsulation, WS = and the remaining cedarwood essential oil in the supernatant after centrifugation).

### Characterization of nanoparticle

The size distribution of the nanocapsules was measured and observed by TEM (Jeol 1011, Japan) at a magnification of 100000×.

### Fourier transform infrared spectroscopy (FTIR)

FTIR analyses for pure pectin, pure CWO and pectin nanoparticles (loaded or unloaded with CWO) were recorded from wave number 500–4000 cm^−1^ by FTIR spectrometer (Burker Tensor 37, Germany) using ATR transmittance mode. Sixteen scans were obtained at a resolution of 4 cm^−1^.

### Thermal properties

The physicochemical compatibility between the cedar wood essential oil and encapsulated polymer were evaluated by DSC analysis. The DSC thermograms (DSC Satarum, France) obtained for oil, encapsulated oil and pectin powder. Samples were heated at a temperature range between 50 and 250 °C at a heating rate of 10 °C min^−1^.

### Coating of capsules on mini bag

Cotton tea bag procured from local supplier (specifications as; fine and lightweight bleached 100%plain-weave 135 g/m^2^, 80 ends per inch × 78 picks per inch) was used for coating. The tea bag was treated according to previously described method^26^. The bag was immersed in nanoencapsulated suspension containing (0.01% W/v) acrylate as binder agent. The mixture containing immersed bags was heated at 30 °C for 1 h. The bags, dried at 50 °C for 5 min and cured at 120 °C for 5 min. Similar concentration (10%) of control and nonencapsulated samples was used to treat bags which was used as reference.

### Surface morphology of coated bag

To study the morphology of bags, SEM imaging was performed. A part of treated bag was mounted on aluminum stubs using a conductive carbon tape and gold palladium sputtered for a conductive coating. A SEM Tescan Vega 3, Czech Republic, was used at 20 kV accelerating voltage to image the surface of the bag. Untreated bag was also imaged for reference.

### Collection and maintenance of *Anopheles culicifacies*

The Laboratory reared larvae of *Anopheles culicifacies* were obtained from National Institute of Malaria Research (NIMR), Insectary Delhi. The larvae were maintained in the standard condition of 25 ± 2 °C and humidity 85 ± 5% kept in tap water in an enamel bowl. Larvae were fed on mixture of dog biscuit and yeast powder in the (3:1) ratio.

### Dose response bioassays on *Anopheles culicifacies*

The comparative larvicidal bioefficacy of coated bag 1(Nanoencapsulated cedarwood in pectin) and bag 2 (Cedar wood nonencapsulated) was evaluated against 3rd instar larvae of *Anopheles culicifacies* following WHO standard larval susceptibility test method^66^. The coated bags were introduced in water (250 mL). Larvae (20 individual) were released into each test container containing. A set up containing water, Tween 80:Span 20 (4:1) served as control. Each experiment was carried out in three replications. The number of deceased larva was counted in the exposed population during the exposure period of 24 h. Corrected mortality was calculated using formulae (WHO 2009).

$$Mortality \left(\%\right)=\frac{\left(X-Y\right)\times 100 }{\left(100-Y\right)}$$

(where *X* = Percentage mortality in treated sample, *Y* = Percentage mortality in control sample).

### Histopathology alterations in larva induced by CWO nanocapsules

To study the mode of action of cedar wood nanoencapsulated on larva, the treated dead larva of Anopheles was studied through histopathology. The mortal larvae was removed from the treated solution and stored in buffered formalin reagent (pH 7.2). The larva was dehydrated by passing through graded ethanol series and embedded in paraffin wax^67^. A thin longitudinal section (LS) of the larva stained with Hematoxylin and Eosin (Hi-Media labs) was cut using microtome (Leica, Germany) and mounted on glass slide. The LS of the midgut region were examined under an Upright Olympus microscope. A setup containing tween, span and water served as control was also performed for reference.

### Statistical analysis

The results of mortality calculated here are means of three replicates. Analysis of variance (ANOVA) was performed on the data and means were compared using Duncan’s multi- range test with SPSS 10.0 software. The significance level was p < 0.05.

## Conclusions

The pectin-based nanocapsules loaded with cedar wood essential oil was developed and characterized. The nanocapsules were impregnated on to mini cotton bag producing ready to use (RTU) formulation to treat mosquito-breeding sites. An important feature of the nanocapsules impregnated bag in larvicidal control program is greater effectiveness with long lasting efficacy and comparative simplicity to the existing control methods. The devised technique may contribute in minimizing risk of dustiness and contamination as the bag can be directly placed in mosquito breeding sites. Moreover, the incorporation of botanical pesticide in the nanocapsules may have added advantages of dose reduction, associated risks due to synthetic pesticides and the long-lasting residual effect. The promising results obtained in the larvicidal bioassays provides basis for the future research under realistic field conditions. Therefore, the technique devised in this work could potentially to be used as an effective larvicide in the mosquito management program, which may contribute towards sustainable vector control strategies and combating against mosquito-borne diseases.

## Supplementary information

Supplementary file1

## Data Availability

All the relevant data are provided in this paper and in Supporting Information files.
