# Supplementary material for: Impregnation of pectin-cedarwood essential oil nanocapsules onto mini cotton bag improves larvicidal performances
Source: Sci Rep. 2020 Aug 24;10:14107. doi: 10.1038/s41598-020-70889-z (PMC7445301; doi:10.1038/s41598-020-70889-z)
Supplement: Supplementary file 1 — Supplementary file1 [file 41598_2020_70889_MOESM1_ESM.pdf]

Impregnation of pectin-cedarwood essential oil nanocapsules onto mini cotton bags improves larvicidal performances

Smriti Kala[1,2*], Nisha Sogan[3], S.N Naik[2] Amrish Agarwal[1], Jitendra Kumar[1]

[1]Formulation Division, Institute of Pesticide Formulation Technology (IPFT), Gurugram- 122016, Haryana, India

[2]Center for Rural Development Technology (CRDT), Indian Institute of Technology (IIT) Delhi-110016, India

[3]National Institute of Malaria Research (NIMR), Delhi-110077, India

Contact details of Authors:

Smriti Kala: smritikl16@gmail.com

S.N Naik: naiksatya581@gmail.com

Nisha Sogan: nisha.singla09@gmail.com

Amrish Agarwal: amrish.ag@hotmail.com

Jitendra Kumar: jitendra.24@gmail.com

***Author for Correspondence:**

Smriti Kala
Scientist (Formulation)
Institute of Pesticide Formulation Technology (IPFT)
(Ministry of Chemicals & Fertilizers, Government of India)
Gurugram - 122016 (Haryana) India
Email: smritikl16@gmail.com, smritikl@yahoo.co.in

Supplementary figure legends

Figure S1: Illustration of pesticides formulation and their handling risks

Figure S 2: Preparation of CWO loaded nanocapsules via ionic gelation and impregnation of nanocapsules on tea bag

Figure S3: Gas chromatogram of cedarwood essential oil along with chemical structures of major compounds of oil A) Alpha Himachlene B) Beta Himachlene C) Atlantone Trans-, Alpha.

Figure S4: Mechanism of adhesion of pectin nanocapsules on cotton fiber in presence of acrylate.

Figure S1:

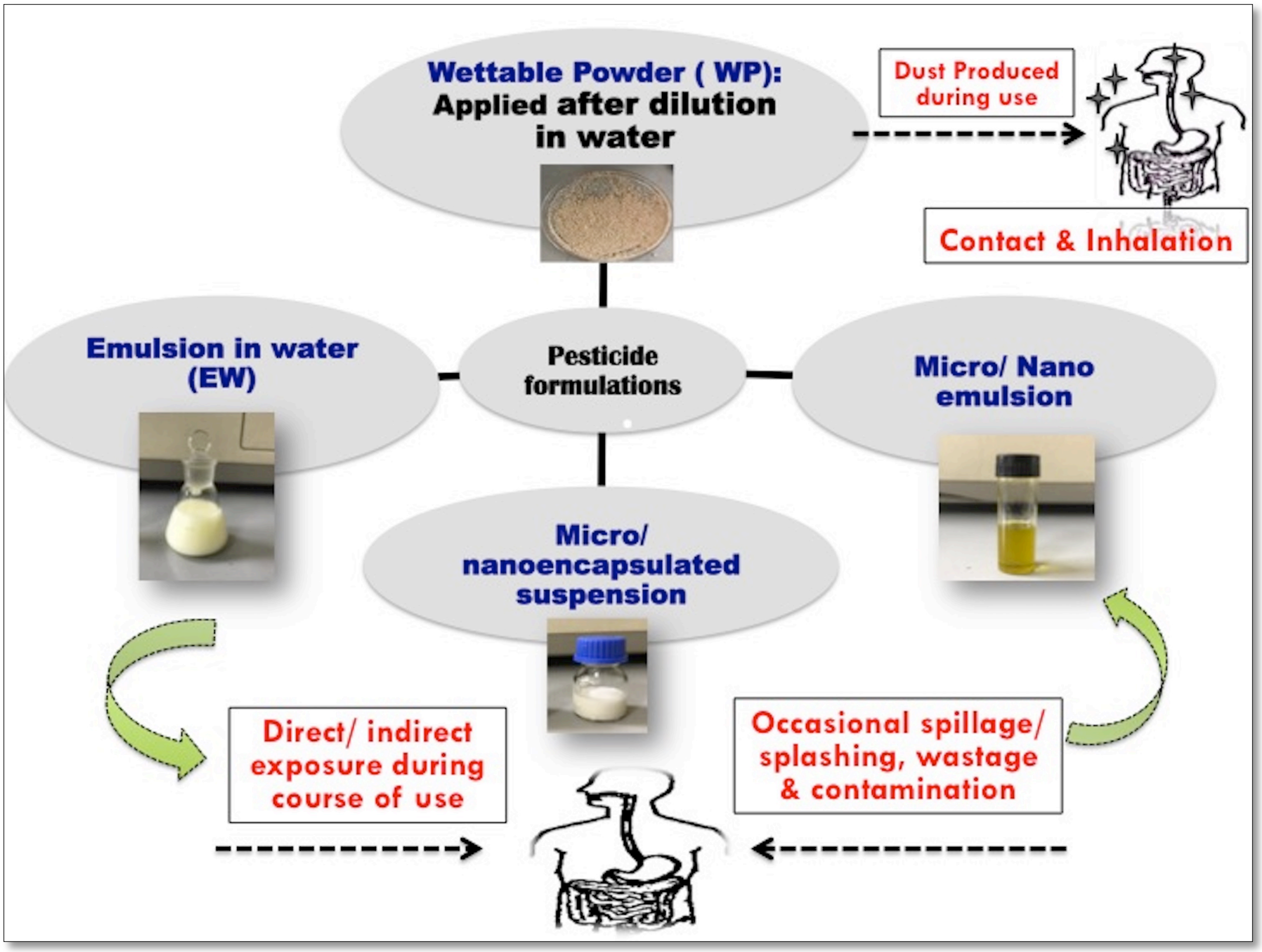

Figure S2

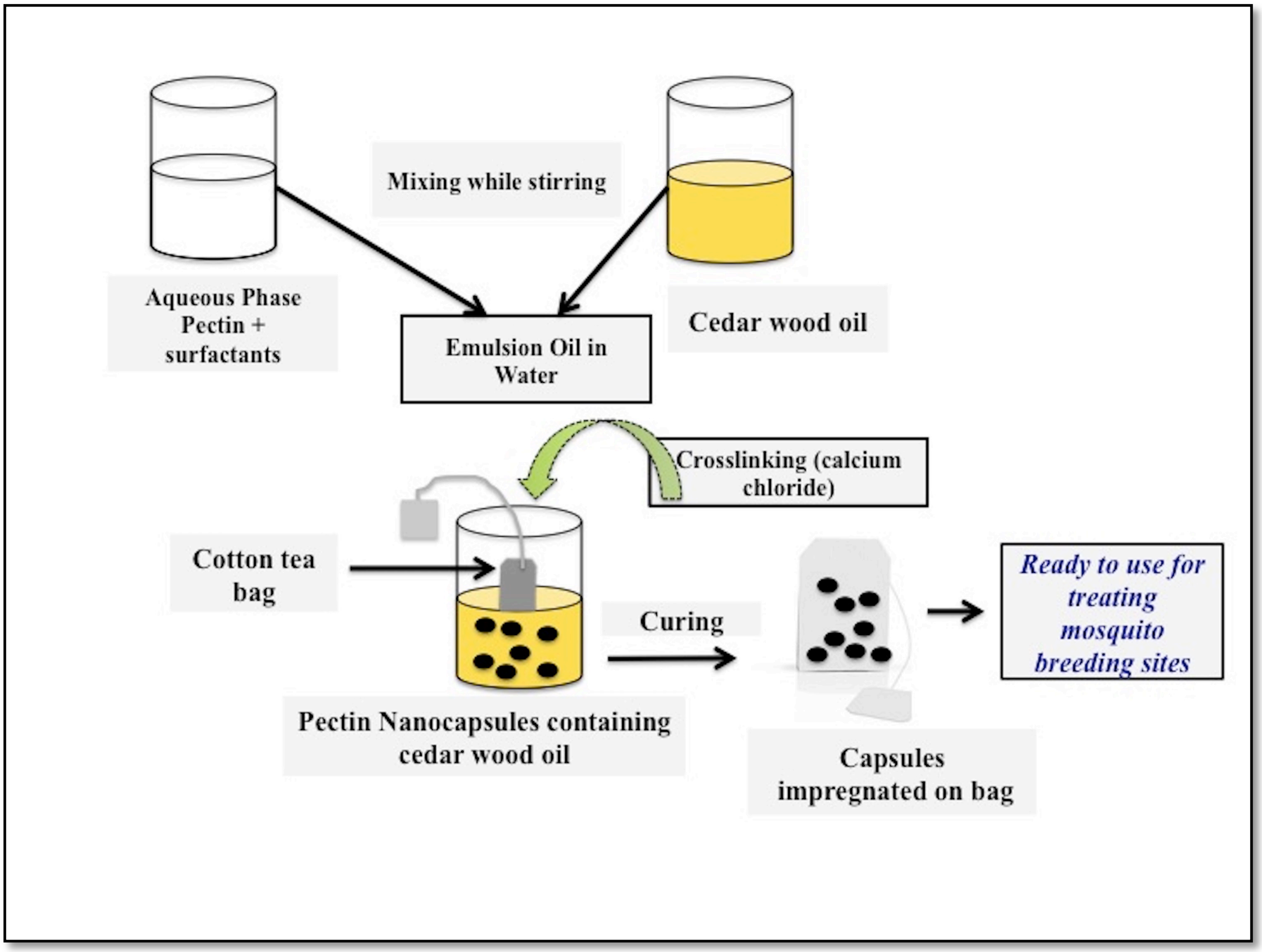
Mixing while stirring
Aqueous Phase
Pectin +
surfactants
Emulsion Oil in
Water
Cedar wood oil
Crosslinking (calcium
chloride)
Cotton tea
bag
Curing
Ready to use for
treating
mosquito
breeding sites
Pectin Nanocapsules containing
cedar wood oil
Capsules
impregnated on bag

Figure S3

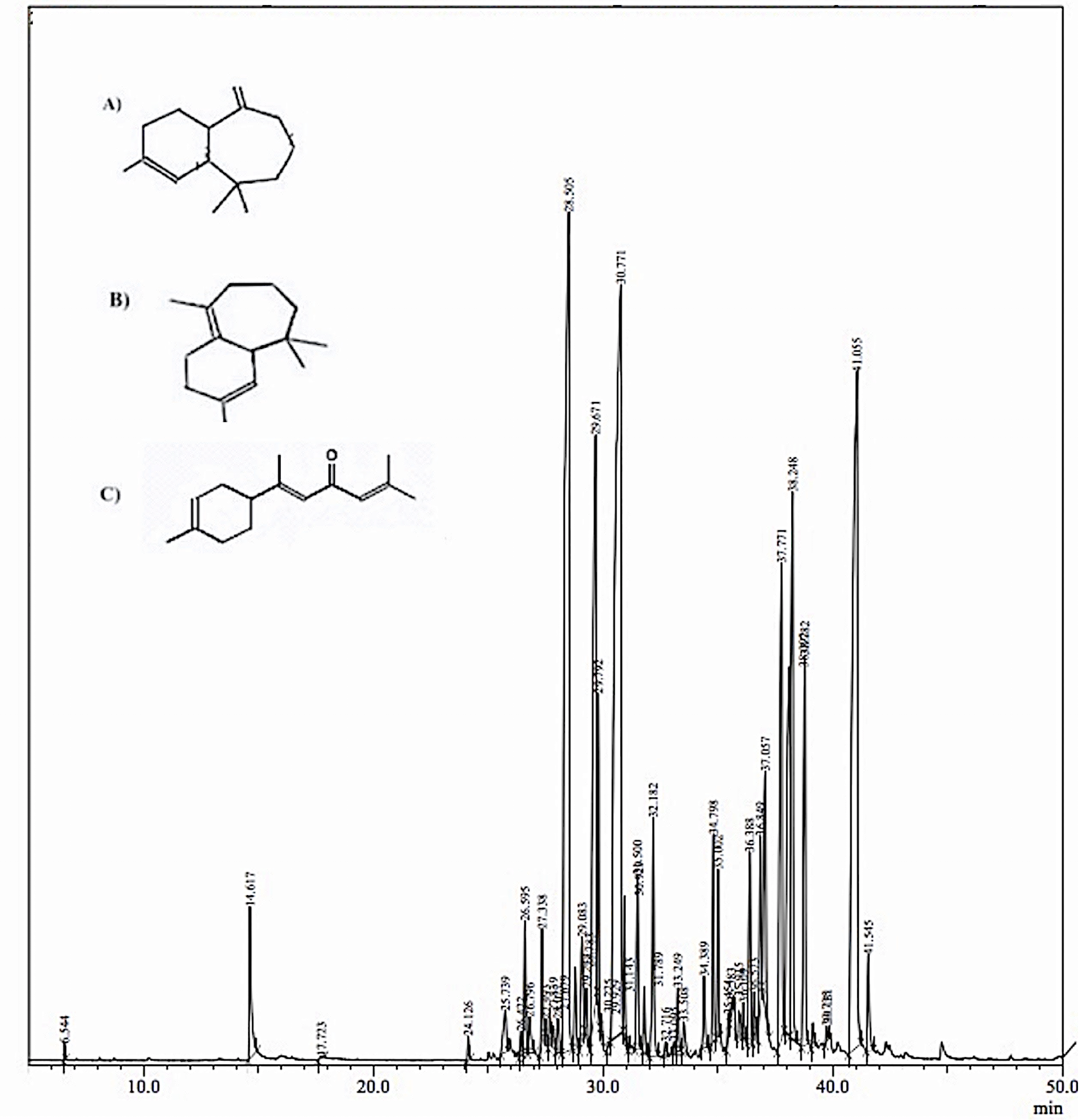
A)
B)
C)
O
6.544
14.617
17.723
24.126
25.739
26.595
27.338
28.505
29.083
29.671
29.792
30.771
32.182
34.339
34.798
36.383
37.057
37.771
38.248
41.055
41.545
10.0
20.0
30.0
40.0
50.0
min

Figure S 4

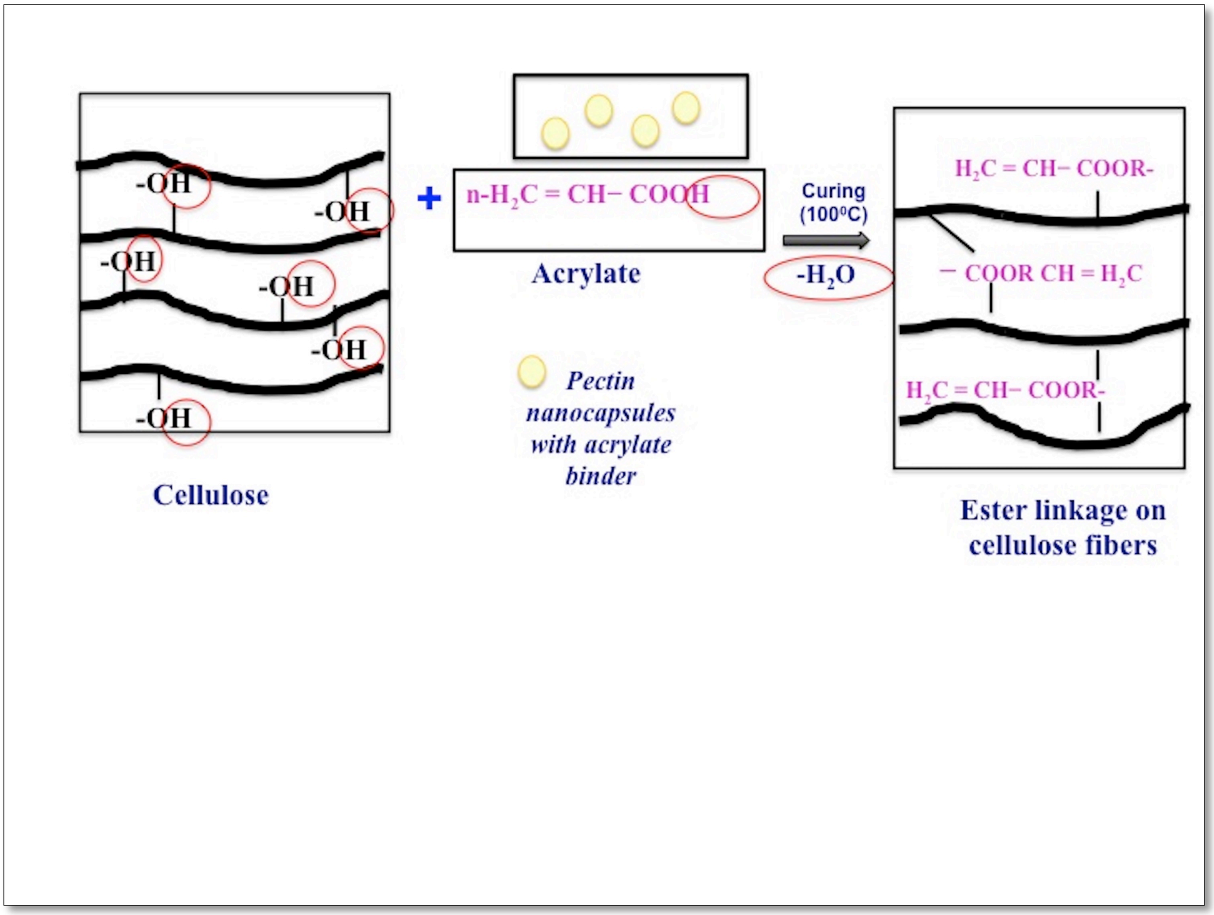
-OH
-OH
-OH
-OH
-OH
-OH
Cellulose
n-H2C = CH− COOH
Acrylate
Pectin nanocapsules with acrylate binder
Curing (100⁰C)
-H2O
H2C = CH− COOR-
− COOR CH = H2C
H2C = CH− COOR-
Ester linkage on cellulose fibers
